# Pre-analytical processing of plasma and serum samples for combined proteome and metabolome analysis

**DOI:** 10.3389/fmolb.2022.961448

**Published:** 2022-12-20

**Authors:** Hagen M. Gegner, Thomas Naake, Aurélien Dugourd, Torsten Müller, Felix Czernilofsky, Georg Kliewer, Evelyn Jäger, Barbara Helm, Nina Kunze-Rohrbach, Ursula Klingmüller, Carsten Hopf, Carsten Müller-Tidow, Sascha Dietrich, Julio Saez-Rodriguez, Wolfgang Huber, Rüdiger Hell, Gernot Poschet, Jeroen Krijgsveld

**Affiliations:** ^1^ Centre for Organismal Studies (COS), Metabolomics Core Technology Platform, University of Heidelberg, Heidelberg, Germany; ^2^ Genome Biology Unit, European Molecular Biology Laboratory (EMBL), Heidelberg, Germany; ^3^ Bioquant, Faculty of Medicine, Institute for Computational Biomedicine, University of Heidelberg and Heidelberg University Hospital, Heidelberg, Germany; ^4^ Faculty of Medicine, University of Heidelberg, Heidelberg, Germany; ^5^ Division Proteomics of Stem Cells and Cancer, German Cancer Research Center (DKFZ), Heidelberg, Germany; ^6^ Department of Medicine V, Hematology, Oncology and Rheumatology, University of Heidelberg, Heidelberg, Germany; ^7^ Center for Mass Spectrometry and Optical Spectroscopy (CeMOS), Mannheim University of Applied Sciences, Mannheim, Germany; ^8^ Division Systems Biology of Signal Transduction, German Cancer Research Center (DKFZ), Heidelberg, Germany

**Keywords:** proteomics, metabolomics, plasma, sample preparation, biomarker

## Abstract

Metabolomic and proteomic analyses of human plasma and serum samples harbor the power to advance our understanding of disease biology. Pre-analytical factors may contribute to variability and bias in the detection of analytes, especially when multiple labs are involved, caused by sample handling, processing time, and differing operating procedures. To better understand the impact of pre-analytical factors that are relevant to implementing a unified proteomic and metabolomic approach in a clinical setting, we assessed the influence of temperature, sitting times, and centrifugation speed on the plasma and serum metabolomes and proteomes from six healthy volunteers. We used targeted metabolic profiling (497 metabolites) and data-independent acquisition (DIA) proteomics (572 proteins) on the same samples generated with well-defined pre-analytical conditions to evaluate criteria for pre-analytical SOPs for plasma and serum samples. Time and temperature showed the strongest influence on the integrity of plasma and serum proteome and metabolome. While rapid handling and low temperatures (4°C) are imperative for metabolic profiling, the analyzed proteomics data set showed variability when exposed to temperatures of 4°C for more than 2 h, highlighting the need for compromises in a combined analysis. We formalized a quality control scoring system to objectively rate sample stability and tested this score using external data sets from other pre-analytical studies. Stringent and harmonized standard operating procedures (SOPs) are required for pre-analytical sample handling when combining proteomics and metabolomics of clinical samples to yield robust and interpretable data on a longitudinal scale and across different clinics. To ensure an adequate level of practicability in a clinical routine for metabolomics and proteomics studies, we suggest keeping blood samples up to 2 h on ice (4°C) prior to snap-freezing as a compromise between stability and operability. Finally, we provide the methodology as an open-source R package allowing the systematic scoring of proteomics and metabolomics data sets to assess the stability of plasma and serum samples.

## Introduction

Mass spectrometry-based metabolomics and proteomics are emerging technologies that are increasingly used in laboratory and clinical settings to refine our understanding of disease biology, vulnerabilities, and resistance mechanisms. Liquid biopsies, such as blood, provide the opportunity to collect information on a patient’s metabolome and proteome status on a longitudinal scale to track disease progression or response to a treatment (Tsonaka et al., 2020; Gummesson et al., 2021). For instance, longitudinal metabolomic profiling of plasma collected from patients suffering from COVID-19 was linked to disease progression, including a panel of metabolites collected at the onset of the disease that may predict the disease severity (Sindelar et al., 2021). Similarly, proteomic analysis of COVID-19 patients revealed protein signatures associated with survival, tissue-specific inflammation, and disease severity (Filbin et al., 2021). The independent analysis of such complex diseases yields promising findings, highlighting that the present technologies are not the limiting factors for the broader use of mass spectrometry (MS) in clinical workflows.

MS-based technologies have matured over the past years, allowing the investigation of analytically challenging but highly informative samples such as blood plasma and serum. Technical advances comprise but are not limited to 1) increased reproducibility and automation in sample preparation; 2) faster, more sensitive, and robust MS instruments; and 3) improved data analysis algorithms, multi-omics, and integrative workflows. While these developments reduce technical noise in the data sets and improve the detection of true biological variability, their efficacy may be compromised if the quality of the starting material is not strictly controlled and standardized.

Although standard operating procedures (SOPs) for blood collection are often in place to suit clinical routine, they may not be harmonized between clinics, and they usually are not optimized to preserve proteins and metabolites for subsequent *omics* analyses. In particular, differences in sample handling (e.g., temperature, sitting time, and use of anticoagulants) may alter the observable protein and metabolite patterns. In biomarker discovery studies, these pre-analytical factors are crucial and have to be considered by clinicians and analysts (Lippi et al., 2020).

Previous studies have highlighted the effects of such pre-analytical factors and often recommend best practices for metabolomic analyses (Yin et al., 2015) or proteomic analyses (Hassis et al., 2015) independently. While either technique already produced a set of potential quality markers related to blood samples, to our knowledge, few studies analyzed the effect sizes of varying pre-analytical parameters in a combined proteomic and metabolomic analysis on the same samples to harmonize the requirements for both techniques with such a comprehensive set of features (Cao et al., 2019). Critically, sample collection and handling requirements differ between metabolomics and proteomics and need to be adjusted accordingly for a combined clinical SOP.

**FIGURE 1:**
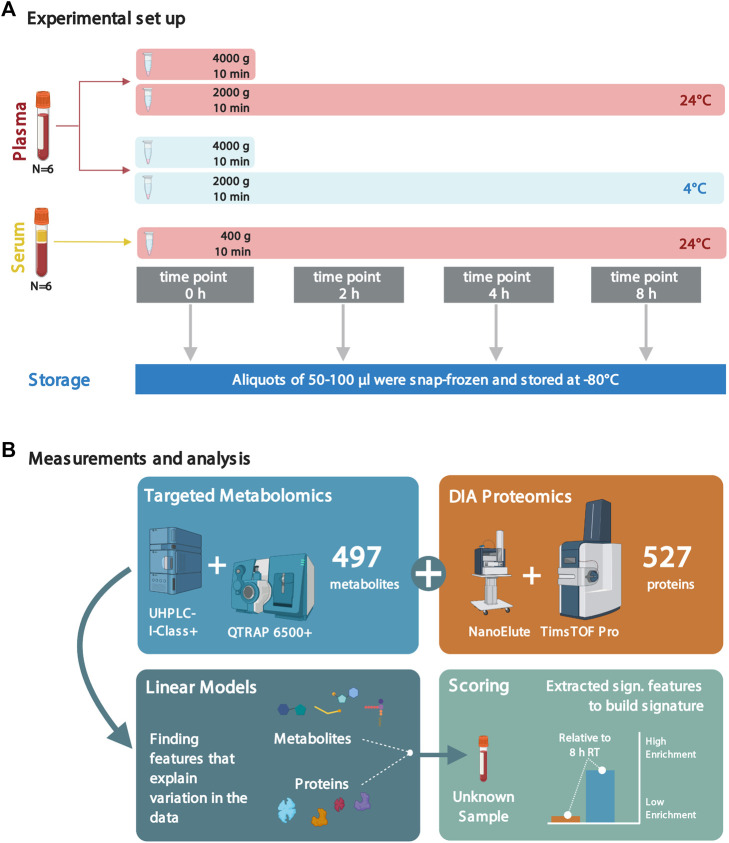
Experimental setup and analyses to assess the effects of sample handling or treatment on metabolite and protein levels. **(A)** Plasma and serum samples from six healthy individuals (all male, in fasting condition; blood collected at the same time of the day in the early morning) were subjected to different sample treatment and handling conditions: sitting times of 0, 2, 4, and 8 h; incubation temperatures of 4°C and 24°C (RT); and two different centrifugal levels (2,000 and 4,000 × g) for the plasma samples. Due to quality considerations, the metabolomics data set only consisted of data from five individuals (see Methods). **(B)** Following the factorial outline, metabolite and protein levels were obtained by mass spectrometry. Next, proteomic and metabolomic data were analyzed using linear models. The significant features were extracted and used to build scores to assess sample quality. Several figures were created with BioRender.com.

Here, we assess how pre-analytical factors impact metabolite and protein levels in plasma and serum samples caused by differences in sitting time, temperature regimes (4°C and room temperature (RT), only RT for serum), and centrifugal acceleration levels. Using targeted metabolic profiling and a single-shot, data-independent acquisition (DIA) proteomics approach, we determine that keeping blood samples on ice (4°C) for up to 2 h prior to snap-freezing are the optimal conditions to preserve metabolites and proteins for a combined metabolomics/proteomics workflow. We introduce an open-source scoring system to assess the quality of plasma and serum samples (Figure 1B).

## Results

To assess how sample handling and treatment affect the stability of protein and metabolite levels in human plasma and serum samples, we selected four time points between centrifugation and snap-freezing to quench samples as follows: 0 h as the baseline of metabolite and protein levels immediately after sampling, 2 h as the clinically feasible time point to quench samples, 4 h as a middle point, and 8 h (quenching at the end of a typical working day) (Figure 1A). Furthermore, samples were kept at different temperatures during these sitting times (on ice/4°C and at RT) to investigate their influence on altering the metabolome and proteome composition. Additionally, we included two centrifugation schemes for plasma samples (2,000 and 4,000 × g). However, we could not attribute any significant changes in the plasma metabolome and proteome between different centrifugation conditions and therefore only applied 2,000 × g in the following sections.

### Identifying metabolites and proteins affected by temperature and sitting time

Analysis of plasma and serum samples by targeted metabolic profiling and a single-shot, data-independent acquisition (DIA) proteomics approach yielded quantitative information for a total of 497 metabolites (Supplementary Datasheet S1) and 572 proteins (Supplementary Datasheet S2). An initial LIMMA analysis (Table 1) showed a high number of features that differed between individual blood donors (*α* < 0.05, after FDR correction using the Benjamini–Hochberg (BH) method). In addition, PCA (Supplementary Figure S1A) of the proteomic and metabolomic data sets indicated individual effects, a finding that was further supported by t-SNE and UMAP analyses (Supplementary Figure S2) and multi-omics factor analysis on both data modalities (MOFA, Supplementary Figure S1A). Especially for the metabolomics data set, there was a clear separation between individuals, while for the proteomics data set, we found less pronounced effects (Supplementary Figure S2D). This analysis suggests that there are dominating individual effects that are reflected in the metabolomics data set and to a lesser extent in the proteome. To gain further insight into this, we next performed classification by partial least square-discriminant analysis (PLS-DA) and sparse PLS-DA (sPLS-DA) to discriminate the individuals based on the metabolite and proteomics profiles. Indeed, it was possible to classify the individuals using the metabolite and protein levels with a low classification error (Supplementary Figure S1C), suggesting that there are features in the metabolomics and proteomics data where individual effects are prevalent. The metabolites and proteins in Supplementary Table S1 were selected by sPLS-DA to explain the variance using the *individual* as the class vector (Supplementary Figure S1B).

**TABLE 1:** Number of features with a significant effect from the factor *individual*. Shown is the number of significant features after FDR correction (*α* < 0.05) for the factor *individual* by LIMMA analysis (see Methods for further details). The total number of features is 497 (metabolites) and 572 (proteins).

	Plasma	Serum
Metabolites	470	435
Proteins	221	151

We also performed PLS-DA to discriminate for *time* and the combination of *time* and *temperature* (Supplementary Figure S4). Both binary classification problems yielded models with higher classification rate errors and lower values for the explained variance for both the proteomics and metabolomics data sets (Supplementary Figure S4A, C) compared to *individual* as the class vector (Supplementary Figure S1). The sPLS-DA analysis yielded a list of features that were used to explain the class vectors of the data and could be regarded as features that change along the *time* and *time*/*temperature* axes (Supplementary Figures S4B, D). We included this list in Supplementary Tables 2, 3, 4.

**FIGURE 2:**
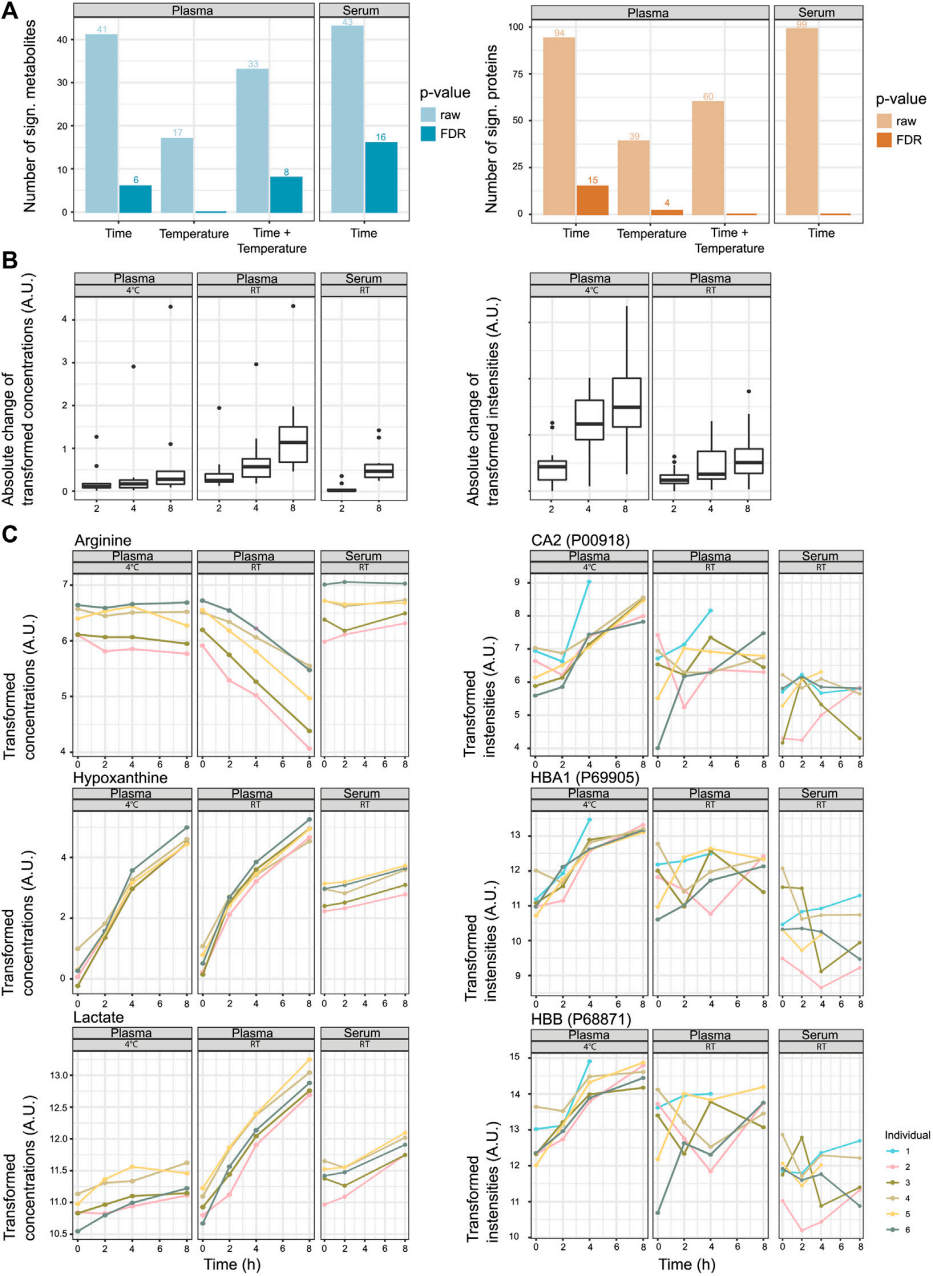
Stability of metabolites and proteins along the sitting time and temperature axes. **(A)** Number of significant metabolites or proteins that show changes according to pre-analytical factors *time*, *temperature*, and the interaction *time/temperature* (raw *p*-values and *p*-values after FDR correction, *α* < 0.05). **(B)** Absolute change of transformed concentrations/intensities of significant metabolites and proteins along the time axis (after FDR correction). The changes to the time points 2, 4, and 8 h are displayed as the mean changes of the individuals (intensity at time point 0 h is 0). For plasma, the features that are significant for the pre-analytical factors *time*, *temperature*, or the interaction *time/temperature* (*α* < 0.05, FDR correction) are included. For serum, the features that are significant to the pre-analytical factor *time* (*α* < 0.05, FDR correction) are included. For the proteomics data set and serum, no features were significant to the pre-analytical factor *time*, and the panel is omitted. **(C)** Examples of metabolites and proteins that show a significant association with the pre-analytical factor *time* (hypoxanthine, lactate, CA2, HBB, and HBA1) or interaction *time/temperature* (lactate and arginine) (*α* < 0.05, after FDR correction).

In the next step, we looked into the changes in metabolite and protein levels when considering inter-individual differences. Motivated by the results of the previous analyses (the initial LIMMA analysis, dimension reduction analysis, PLS-DA, and MOFA), which showed that metabolome and proteome variation is influenced by inter-individual differences, we decided to use mixed linear models to determine the features that will change according to sitting time, temperature, or a combination of time and temperature. We modeled as fixed effects *time*, *temperature*, and the interaction terms *time/temperature* (plasma) and *time* (serum). The information on the blood donor (*individual*) was included for both groups as a random effect (Figure 2A). An overview of the absolute change of the significant metabolites and proteins can be found in Figure 2B (see also Figure 2C for exemplary metabolites and proteins). We provide the metabolite- and protein-associated *p*-values for plasma and serum samples in Supplementary Information.

Looking at metabolomics- and proteomics-specific differences, the analysis revealed that metabolite concentrations were less stable at RT, while protein abundances were less stable at 4°C (Figure 2 and Supplementary Figure S5). For the affected features, the absolute change was, in most cases, more prominent after 8 h than after 2 h, yet they were not significant (Supplementary Figure S5).

### Scoring plasma and serum sample quality using proteomic and metabolomic signatures

**FIGURE 3:**
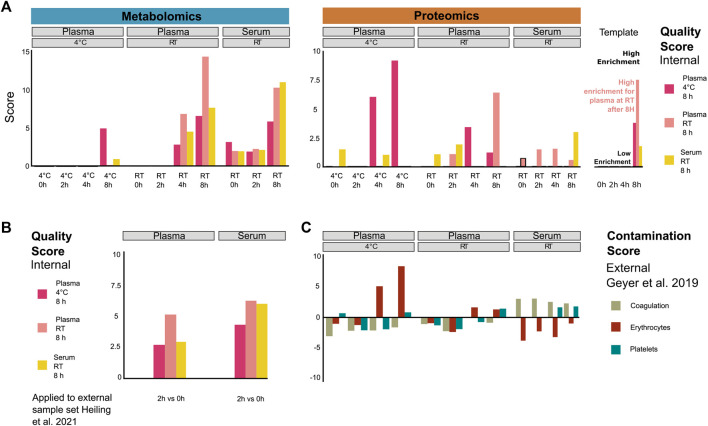
Systematic evaluation of sample quality and contamination with proteomic and metabolomic signatures. **(A)** Proteomic/metabolic pre-analytical condition signature scores for each sample group (as averaged from individual samples). Signature scores are normalized enrichment scores, representing the number of standard deviations away from the mean of an empirical null distribution of scores. A high score means that the sample displays an enrichment of markers of the corresponding signature compared to other samples. While the scores generated can be both positive and negative, we focus exclusively on positive scores in this figure. **(B)** Metabolic pre-analytical signature applied to score samples from an external study (Heiling et al., 2021). **(C)** Coagulation, erythrocyte, and platelet signatures were used to score the contamination of proteomic plasma and serum samples. Signatures were taken from Geyer et al. (2019).

We next investigated whether patterns of potential protein and metabolite deregulation (with respect to *time* and *temperature*) could be used as a quality metric for samples obtained under the tested conditions. We selected the top 20 proteins and metabolites ranked by *p*-value to generate signatures under the following handling conditions: plasma kept on ice (4°C) or RT for 8 h and serum at RT for 8 h (Supplementary Figure S6). While it may be difficult to draw conclusions regarding the significance of individual metabolites or proteins due to the limited sample size, their combined signal may hold enough information to score the relative quality of samples with respect to sitting time and temperature. Thus, to confirm that these signatures could yield sensible insight into sample integrity, we computed an average normalized enrichment score (NES; see Methods) for each signature under the respective sample pre-analytical conditions. If the signatures are indeed informative, we should expect to observe a steady increase in the enrichment of the respective signature for each condition. Coherently, each signature showed higher scores for samples that matched the respective conditions from which they were derived (Figure 3A). This notably showed that the plasma protein signature at RT over time already scored highly in samples stored at RT for 4 h, as opposed to the signature of plasma/4°C/8 h which only scored highly in the samples obtained at low temperature and after 8 h, as expected. This pattern was inverted for the metabolic signatures of plasma samples. This indicates that the changes are more pronounced at the metabolomic level when samples are stored at 4°C than those at RT, while changes are more pronounced at the proteomic level for plasma samples kept at RT.

Finally, while the NES can take both positive and negative values, here, we focused only on the positive values to simplify the interpretation of the results. Since the data were normalized in a way that each measurement is scaled relative to other samples of the cohort, the scores will be drawn from a distribution where an NES of 0 represents samples that have average levels of degradation compared to the overall cohort, and any value above that represents samples that show higher degradation than the rest of the cohort. It is worth noting that this scoring can only score samples in a manner relative to the rest of the cohort and cannot provide absolute quantification of sample degradation. In order to get the most out of such a method, it is advised to always include at least one reference sample of known quality.

To validate this approach, we used the signatures to score metabolomic results from an external study (Heiling et al., 2021), where plasma and serum samples were kept at RT for 2 h (Figure 3B). The plasma/RT/8 h metabolic signatures got a higher score (NES = 5.1) than plasma/4°C/8 h (NES = 2.6) and serum/RT/8 h (NES = 2.9) signatures. However, the serum/RT/8 h signature score was similar to the plasma/RT/8 h signature score (NES = 6.1 and NES = 6.4, respectively). Thus, the serum RT/8 h and plasma/RT/8 h metabolic signatures appear less discriminant than the plasma/4°C/8 h metabolic signature. Nevertheless, the best scores overall matched the actual experimental conditions that were used, indicating that the scoring system holds up beyond the data set that was used for training.

In order to further characterize the changes that we observed in the plasma and serum samples over time, we investigated if proteomic signatures could be associated with contamination by proteins originating from specific blood cells. We obtained proteomic markers of coagulation, erythrocyte, and platelet contamination from Geyer et al. (2019). Plasma samples kept on ice (4°C) for 4 h and 8 h showed the highest enrichment of erythrocyte contamination markers (Figure 3C), mainly driven by CAT, CA2, BLVRB, PRDX2, and ALDOA (Supplementary Figures S6A, B). Interestingly, the plasma/4°C/8 h signature seems to also be specifically driven by a lower abundance of the VWF protein, a blood glycoprotein involved in platelet adhesion (Supplementary Figure S6C). The contamination scores were lower in plasma samples than those that were kept at RT, although they still showed a progressive increase over time. On the other hand, serum samples exhibited no significant increase in erythrocyte contamination score, instead showing a consistently high (albeit slightly decreasing) score for coagulation markers over time, as expected. This signature was mainly driven by increased PPBP and THBS1 and decreased F13A1 (Supplementary Figures S6A, D). In a similar fashion, we displayed the main drivers of the metabolomic-derived signatures such as hypoxanthine, lactate, ornithine, and aspartate (Supplementary Figures 6A, E, F, G). Taken together, these results show that scoring provides a quantitative metric for the quality control for proteomic and metabolomic data of plasma and serum samples. This should be a helpful tool to exclude low-scoring samples for further analysis.

## Discussion

The progress of MS-based technologies over the past years has enabled the characterization and quantification of analytically challenging but clinically accessible samples such as blood plasma and serum. Although SOPs for individual metabolic and proteomic analyses have been developed (Pasella et al., 2013; Yin et al., 2015; Tuck et al., 2019; Lippi et al., 2020), there is no consensus on their combined application for the molecular characterization of blood samples in a clinical setting.

Here, we performed a comprehensive analysis on the stability of 497 and 572 metabolites and proteins in blood plasma and serum to scrutinize the effects of various treatment regimens (sitting time and incubation at different temperatures) to simulate different sample handling scenarios. Notably, our experiment aimed to define an SOP trade-off regarding the different requirements for metabolomics and proteomics to effectively apply both approaches to the same blood samples. In addition, we aimed to implement objective quality scoring as a metric for sample quality and potential contamination. Although this study was performed on a small cohort of healthy volunteers, the findings have implications for the sampling procedure of clinical blood collection and the bioinformatics analysis for quality control.

### Measuring the change of the metabolome and proteome

**FIGURE 4:**
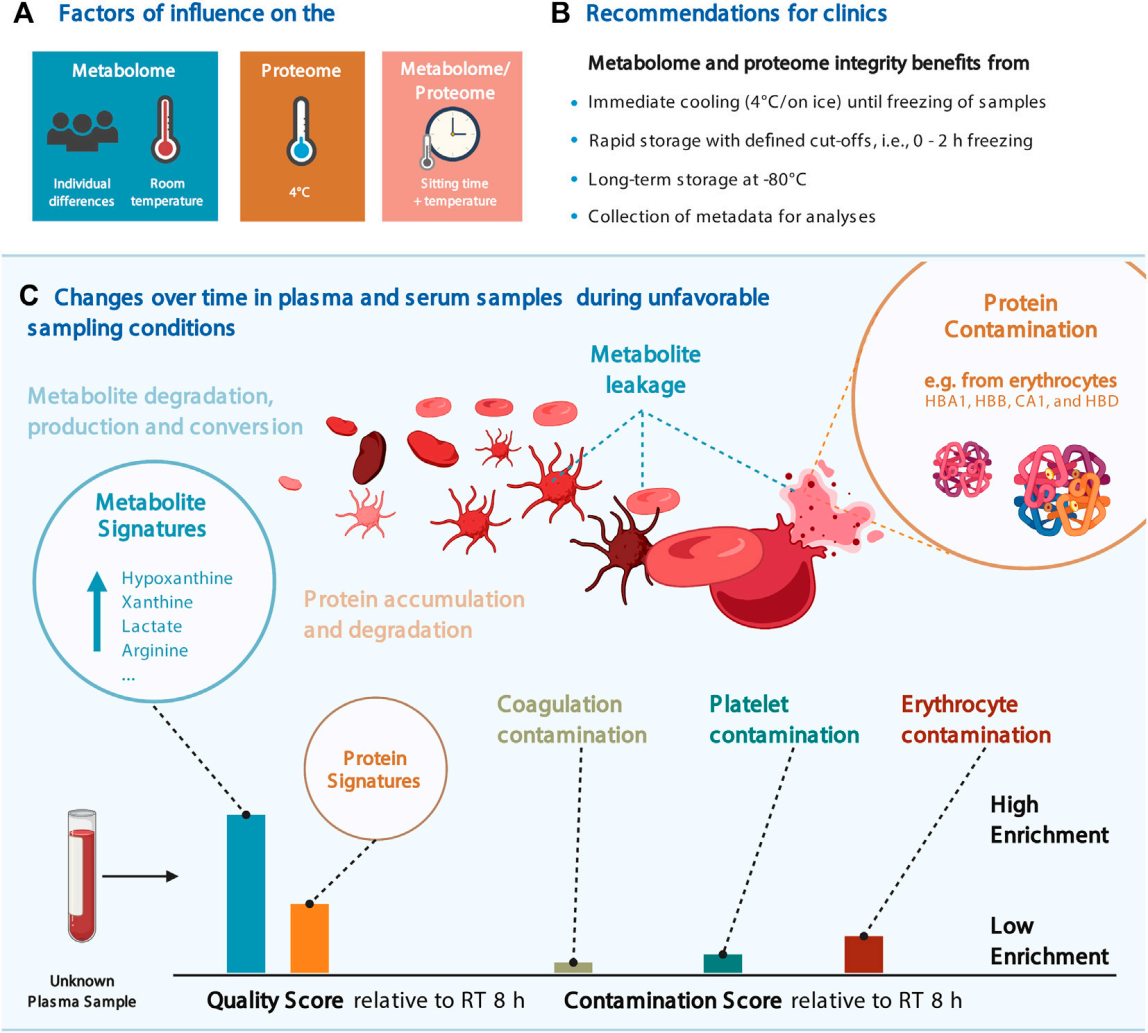
Considerations for the joint proteomic and metabolomic analysis of plasma and serum samples. **(A)** Most influential factors on the proteome and proteome in our data. **(B)** Recommendations for clinical blood sampling. **(C)** Change over time in blood plasma and serum samples and the resulting quality and contamination scores. Several figures were created with BioRender.com.

Through our statistical analyses, we detected changes in numerous features that may affect the biological interpretation of clinical metabolomics and proteomics data sets. Some metabolites (e.g., hypoxanthine, xanthine, lactate, arginine, ornithine, and cystine) and proteins (e.g., CA1, CA2, HBB, HBD, and HBA1) showed a profound dependency on sitting time and temperature (or a combination thereof) (Figure 2 and Figure 4A).

Metabolite classes such as amino acids, purines, and carbohydrates vary in abundance and are therefore usually investigated to answer biological questions. Also, these well-known metabolites affected by the conditions represent only 10% of our data set, and we have increased the panel of temperature- and time-sensitive metabolites due to the broad metabolite coverage. In fact, lipids are the largest observed class of metabolites, most of which were stable across the tested conditions (Supplementary Material S1).

Temperature and time are well known to affect metabolite and protein levels (Kamlage et al., 2014; Cao et al., 2019; Daniels et al., 2019; Stevens et al., 2019). Elevated levels of hypoxanthine and amino acids over time (Ferreira et al., 2019) and the deregulation of cholesterol metabolism were also previously documented (Ryu et al., 2016). The association of these and other metabolites to a pathological condition, therefore, needs to be evaluated with caution to exclude the possibility that they emerge inadvertently by sample handling or technical bias. Strict pre-analytical measures help to gain confidence in the subsequent biological and clinical interpretation based on the measured features.

For most of the features in our data sets, we only found minor changes under the experimental conditions applied (Figure 2). Then, 90% of metabolites and 97% of proteins only varied slightly over time. Other metabolome studies reported similar proportions where 91% of the metabolite remained stable over several pre-analytical conditions (Ferreira et al., 2019). This implies that in clinical research studies where large effect sizes from biological differences are known or expected and large cohorts are used, the contribution to feature-level variation stemming from sample handling might be partially alleviated. The integration of several data sets, that is, proteomic and metabolomic, from the same sample may also mitigate bias.

### Rapid handling and cold storage for up to 2 h as SOP

Although the increased stability of the metabolome at 4°C was expected, we observed a contrary effect for proteins, showing higher variance at low temperature (Figures 2, 3A), suggesting that proteomics and metabolomics require different pre-analytical conditions to obtain optimal results. Therefore, we propose that keeping plasma and serum samples on ice for up to 2 h is an acceptable trade-off to maintain adequate stability of both the proteome and metabolome (Figure 2 and Supplementary Figure S5). In addition, this should be a condition that can be met in clinical practice (Figure 4B).

Similarly, considerations may be made regarding the storage time of samples in biological repositories such as biobanks. Previous studies showed that long-term storage at −80°C over 7 years only introduces minimal variation and that significant changes occur upon longer storage times (Wagner-Golbs et al., 2019). This highlights the potential to address clinical questions using metabolomics and proteomics from biological repositories under the prerequisite that the sampling collection is comparable. While biobank samples are an essential resource for discovery studies, prospective samples enable the enforcement of SOPs during collection that are more suitable for metabolomic analyses, e.g., by storing samples at 4°C for under 2 h and then quenching by snap-freezing in liquid nitrogen.

### Quality control signatures to score plasma and serum samples

Designing formal criteria for data curation and analysis is crucial to ensuring data robustness. To this end, we devised a scoring system using the significantly altered proteins and metabolites as signatures to evaluate the impact of pre-analytical conditions on the proteome and metabolome integrity of a given sample. Provided as an R package (https://github.com/saezlab/plasmaContamination), this tool can be used for quality control after pre-analytical handling, and in addition, the proteome signatures enable to distinguish the severity and the source of contamination, that is, from platelets, erythrocytes, or resulting from coagulation (Figures 3C, 4C). We showed that the changes in protein abundance in samples stored on ice were mainly related to protein markers of erythrocytes in plasma samples, likely resulting from hemolysis occurring under this condition (Figure 4C). As expected, coagulation signatures scored exclusively high in serum samples.

Both the scores for metabolite and protein contamination enable the quality assessment of plasma and serum samples of unknown origin. Notably, the erythrocyte, platelet, and coagulation signatures were obtained from a large external cohort of samples (>70 samples), while the signatures derived from our own samples were estimated from a comparatively small number of samples (*n* = 6). Although this may affect their discriminative power, the derived signatures and bioinformatics tools are publicly accessible and can therefore be updated and expanded easily when more data become available. The high similarity of the scores for the plasma/4°C/8 h and erythrocyte signatures strongly suggests that the number of samples used in this study (*n* = 6) already allows to generate signatures that are comparable to those generated with a much larger number of samples (>70). While the study could benefit from a larger sample size to generate signatures, important pre-analytical factors can already be scored in a consistent manner with those signatures. Thus, those signatures yielded coherent scores when they were tested with our own samples and validated with samples from an external study. The expansion of such signatures to other pre-analytical factors, such as storage conditions, enables the development of further quality control metrics. This may be achieved through similarly structured experimental set-ups with small sample sizes or the analysis of bigger cohorts with the inclusion of metadata. We anticipate that a quality score for proteome and metabolome integrity can have great practical utility by enabling the exclusion of low-scoring samples for further analysis. This will be particularly important if clinical decisions are to be made based on metabolic or proteomic data from such samples. At this point, it is premature to suggest a cut-off score here, since the number of samples in our study is low and since the choice for such a cut-off may depend on the setting of the analysis (e.g., biomarker discovery and clinical decision). Finally, although a quality score is helpful, it cannot replace rigorous SOPs. In addition, this must be evaluated in the context of other available metadata that should be applied in combination with other quality control strategies (Naake and Huber, 2022).

### Summary

In this study, we assessed the influence of controllable pre-analytical parameters on protein and metabolite levels in plasma and serum samples to define or improve SOPs for concerted metabolomic and proteomic analyses. While only a subset of metabolites and proteins changed, the ability to identify features that are prone to alteration increases the confidence in such broadly acquired data sets. We propose to store blood samples for a maximum of 2 h on ice (4°C) before quenching the samples, as a compromise between stability and practical operability. Additionally, the metabolomic and proteomic signatures can be routinely applied in bioinformatics workflows to review and evaluate the sample quality of plasma and serum samples. Due to their accessibility, such signatures may be expanded over time to improve the assessment of qualitative differences between blood samples. Lastly, bigger sample sizes and additional metadata of volunteers and/or available metadata from clinics may extend these scores to include signatures capturing other sources of variability that are important to clinical studies, such as storage, medication, or lifestyle.

## Methods

### Sampling and sample treatment/design

Peripheral blood samples were collected from six healthy male volunteers (aged 22–37 years, median age was 29.83 years) with written informed consent in accordance with the Declaration of Helsinki and approved by the Ethics Committee of the Medical Faculty of the University of Heidelberg (S-254/2016). All samples were taken in the early morning in a fasting condition using serum and EDTA S-Monovette tubes (Sarstedt AG, Nümbrecht, Germany). Serum samples were allowed to coagulate for 30 min after collection, kept at RT for the indicated time points, and centrifuged at 400 x g for 10 min. Plasma samples were kept at RT or at 4°C/on ice for the indicated time points, followed by centrifugation for 10 min at 2,000 x g and 4,000 x g, respectively. Subsequently, samples were divided into single-use aliquots, snap-frozen in liquid nitrogen, and stored at −80°C until analysis. Thus, metabolomic and proteomic analyses were performed from the same original samples.

### Metabolomics

For the metabolomics analysis of up to 630 metabolites, the Biocrates MxP^®^ Quant 500 kit (Biocrates, Austria) was used following the manufacturer’s protocol. Briefly, 10 µl of plasma or serum was semi-automatically pipetted onto a 96-well plate containing internal standards using a pipetting robot (epMotion 5057, Eppendorf, Germany) and subsequently dried under a nitrogen stream using a positive pressure manifold (Waters, Germany). Afterward, 50 µl of 5% phenyl isothiocyanate (PITC) was added to each well to derivatize amino acids and biogenic amines. After 1 h of incubation at RT, the plate was dried again. To resolve all extracted metabolites, 300 µl of 5 mM ammonium acetate in methanol were pipetted to each filter and incubated for 30 min. The extract was eluted into a new 96-well plate using positive pressure. For the LC-MS/MS analyses, 150 µl of the extract was diluted with an equal volume of water. Similarly, for the FIA-MS/MS analyses, 10 µl of the extract was diluted with 490 µl of FIA solvent (provided by Biocrates). After dilution, LC-MS/MS and FIA-MS/MS measurements were performed in the positive and negative modes on subsequent days. For chromatographic separation, an UPLC I-Class PLUS (Waters, Germany) system was used, coupled with a QTRAP 6500+ mass spectrometry system (Sciex, Germany) in an electrospray ionization (ESI) mode. Data were recorded using the Analyst Software suite (version 1.7.2, Sciex, Germany) and transferred to Met*IDQ* software (version Oxygen-DB110-3005, Biocrates, Austria), which was used for further data processing, that is, technical validation, quantification, and data export.

Low-abundant metabolites that were not measured in more than 66% of the samples at 10 times the levels over the limit of detection (LOD) or above the lower limit of quantification (LLOQ) (both according to MetIDQ software) were removed from the subsequent analysis. Using the MatrixQCvis package (Naake and Huber, 2022; version 1.1.0), low-quality samples were removed, leading to the exclusion of all samples belonging to individual 1 from the further analysis. To prepare the data sets for statistical analysis, the raw intensity values were transformed *via* vsn (vsn2 function from the vsn package, version 3.59.1), which yields a matrix with feature values being approximately homoskedastic (features have constant variance along the range of mean values). Missing values were imputed *via* the impute.MinDet function (imputeLCMD package, version 2.0). While for MOFA analysis, the transformed data set was used, for all other analyses (dimension reduction, PLS-DA, LIMMA analyses, and mixed linear models), the imputed data set was used.

### Proteomics

Sample preparation: Plasma and serum aliquots were diluted 1:10 in ddH_2_O to perform a bicinchoninic acid assay (BCA, Pierce–Thermo Scientific) for protein quantification. Subsequently, 10 μg protein per sample was further processed in a 1 μg/μl concentration and 100 mM ammonium bicarbonate (ABC, Sigma-Aldrich) using a Covaris LE220-plus focused-ultrasonicator for AFA-based ultrasonication in a 96-well format. The plate was transferred to a Bravo pipetting system (Agilent Technologies, United States) for autoSP3 processing as described elsewhere (Müller et al., 2020). In brief, 10 mM TCEP, 40 mM chloroacetamide (CAA), 100 mM ABC, and 1x protease inhibitor cocktail (PIC, complete, Sigma-Aldrich) were added to each sample, followed by incubation at 95°C for 5 min. Protein binding to Sera-Mag SpeedBeads (Fisher Scientific, Germany) was induced by increasing the buffer composition to 50% acetonitrile (ACN, Pierce–Thermo Scientific). The bead stock was prepared as follows: 20 μl of Sera-Mag SpeedBeads A and 20 μl of Sera-Mag SpeedBeads B were combined and rinsed with 1 × 160 μL ddH_2_O and 2x with 200 μl ddH_2_O and re-suspended in 20 μl ddH_2_O for a final working stock. The bead stock was prepared for all samples. The autoSP3 protein clean-up was performed with 2x ethanol (EtOH, VWR International GmbH, Germany) and 2x ACN washes. Reduced and alkylated proteins were digested on beads and overnight at 37°C in a lid-heated PCR cycler (CHB-T2-D ThermoQ, Hangzhou BIOER Technologies, China) in 100 mM ABC with sequencing-grade modified trypsin (Promega, United States). Upon overnight protein digestion, each sample was acidified to a final concentration of 1% trifluoroacetic acid (TFA, Biosolve Chimie). MS injection-ready samples were stored at −20°C.

Data acquisition: Peptide samples were measured using a timsTOF Pro mass spectrometer (Bruker Daltonics, Germany) coupled with a nanoElute liquid chromatography system (Bruker Daltonics, Germany). Peptides were separated using an analytical column (Aurora Series Emitter Column with CSI fitting, C18, 1.6, 75 μm × 25 cm) (Ion Optics, Australia). The outlet of the analytical column with a captive spray fitting was directly coupled to the mass spectrometer using a captive spray source. Solvent A was ddH_2_O (Biosolve Chimie), 0.1% (v/v) FA (Biosolve Chimie), and 2% acetonitrile (ACN) (Pierce, Thermo Scientific), and solvent B was 100% ACN in ddH_2_O and 0.1% (v/v) FA. The samples were loaded at a constant maximum pressure of 900 bar. Peptides were eluted *via* the analytical column at a constant flow rate of 0.4 μL per minute at 50°C. During the elution, the percentage of solvent B was increased in a linear fashion from 2 to 17% in 22.5 min, from 17 to 25% in 11.25 min, from 25 to 37% in a further 3.75 min, and then to 80% in 3.75 min. Finally, the gradient was finished after 3.75 min at 80% solvent B. Peptides were introduced into the mass spectrometer *via* the standard Bruker captive spray source at default settings. The glass capillary was operated at 3,500 V with a 500-V end plate offset and 3 L/min dry gas at 180°C. Data were acquired in a data-independent acquisition (DIA) mode using full-scan MS spectra with a mass range m/z of 100–1,700, and a 1/k0 range of 0.6–1.6 V*s/cm^2^ with 100 ms ramp time were acquired with a rolling average switched on (10x). The duty cycle was locked at 100%, and the TIMS mode was enabled. All timsTOF Pro and nanoElute methods were considered defaults provided by Bruker. Data were acquired in data‐independent acquisition (DIA) mode.

DIA method details: For the DIA scans, resolution was set to 30,000 FWHM, with an automatic gain control (AGC) target of 3 × 10^6^ ions, a fixed first mass of 200 m/z, a stepped collision energy of 27, and a loop count of 34 with an isolation window of 24.3 m/z.

Data processing: Raw files were processed in Biognosys Spectronaut version 14.11. The search parameters were set to default as specified by the developer of the software. In brief, the enzyme was set to trypsin/P with up to two missed cleavages. Carbamidomethylation (C) was selected as a fixed modification; oxidation (M) and acetylation (protein N-term) were set as variable modifications.

Data quality was checked by the MatrixQCvis package (Naake and Huber, 2022, version 1.1.0), leading to the exclusion of several low-quality samples. Further data processing was carried out according to the metabolomics data set using the vsn transformation and imputation of missing values by the impute.MinDet function.

### LIMMA analysis to test for *individual* effects

For the metabolomics and proteomics data sets, the transformed intensities were taken. Separately for the plasma and serum samples, a linear model was fitted to the data (using lmFit from limma, version 3.50.0). For plasma samples, information on the *individual*, *time*, *temperature*, and the interaction between *time* and *temperature* were included as terms in the model. For serum samples, information on the *individual* and *time* were included as terms in the model. t-statistics and moderated F-statistics were computed by empirical Bayes moderation of the standard errors toward a global value (using eBayes from limma). The corresponding *p*-values to the effects for all individuals were adjusted *via* the FDR using the Benjamini–Hochberg method (α < 0.05). The code for the analysis can be found here: https://github.com/tnaake/SMARTCARE_preanalytical_processing/tree/main/LIMMA.

### Multi-omics factor analysis (MOFA) of the combined metabolomics and proteomics data sets

The vsn-transformed data sets (no imputed missing values) were used for running MOFA. For the proteomics data set, the mean intensities between duplicates were calculated. Subsequently, only the overlapping samples (intersection) of the metabolomics and proteomics data sets were retained (40 plasma samples and 14 serum samples). MOFA (using the MOFA2 package, version 1.1.21) was run using the metabolomics and proteomics data sets as views and plasma and serum as groups. The data options were set to default values (scale_views = FALSE, scale_groups = FALSE, center_groups = TRUE, and use_float32 = FALSE), the model options were set to default (Gaussian likelihood for views, maximum number of factors = 15, spikeslab_factors = FALSE, spikeslab_weights = TRUE, ard_factors = TRUE, and ard_weights = TRUE), and the training options were set to default (maximum of iterations = 10,000, convergence mode = “slow,” drop_factor_threshold = 0.01, startELBO = 1, freqELBO = 5, stochastic = FALSE, gpu_mode = FALSE, seed = 42, and weight_views = FALSE). The code for the analysis can be found here: https://github.com/tnaake/SMARTCARE_preanalytical_processing/tree/main/MOFA/.

### Dimension reduction analysis

The dimensions of the metabolomics and proteomics data sets were reduced to two/three dimensions using principal component analysis (PCA), t-distributed stochastic neighbor embedding (t-SNE), and Uniform Manifold Approximation and Projection (UMAP). Prior to performing PCA, the transformed and imputed intensity values were feature-wise scaled and centred before calculating PCs using prcomp (from the stats package, version 4.1.0). t-SNE was run using the Rtsne function with the following parameters: initial dimensions = 10, maximum number of iterations = 100, final dimensions = 3, and perplexity = 3 (Rtsne package, version 0.15). UMAP was run using the umap function with the following parameters: minimum distance = 0.1, number of neighbors = 15, and spread = 1 (umap package, version 0.2.7.0).

### Partial least square-discriminant analysis

To discriminate the samples based on the class vector Y, partial least square-discriminant analysis was performed. Here, Y is a vector of length n that indicates the class of each sample, that is, a vector containing information on the *time*, *temperature*, *time*/*temperature*, or the *individual* identifier. X is an n x p matrix containing the normalized + transformed + imputed intensities. To find the optimal number of components, plsda from the mixOmics package (version 6.15.45) was run with a maximum of 20 components (ncomp = 20), followed by the evaluation of the performance of the fitted PLS using the perf function (validation = “Mfold,” folds = 3, and nrepeat = 30). The overall classification error rate was taken as a measure to select the number of components, and the number of components and distance method were selected by the maximum of the determined component number of the distances “centroids.dist,” “Mahalanobis.dist,” and “max.dist.” In the next step, the optimal number of variables was determined using a grid-based search ranging from 5 to 100 variables by the tune.splsda function (number of components, ncomp, and distance method, dist, as previously determined by the perf function, validation = “Mfold,” folds = 3, nrepeat = 30, and measure = “BER”). The final model, using the optimal number of components based on t-tests on the error rate and the corresponding number of selected variables, was selected using the splsda function (scale = TRUE). All functions were taken from the mixOmics package (version 6.15.45). The code for the analysis can be found here: https://github.com/tnaake/SMARTCARE_preanalytical_processing/tree/main/MLM/metabolomics.

### Stability analysis using mixed linear models

For the mixed linear model, for plasma samples, the *time*, *temperature*, and the interaction between *time* and *temperature* were included as fixed effects and individual as a random effect into the model. For serum samples, *time* was included as a fixed effect and individual as a random effect. When fitting the actual model, the lmer function from the lmerTest package (version 3.1–3) was used. If the mixed linear model was singular, an analysis of variance model was fitted with the same terms as for the mixed linear model except for the random effects. The corresponding *p*-values to the fixed effects (*time*, *temperature*, and interaction between *time* and *temperature*) and the intercept were adjusted *via* the FDR using the Benjamini–Hochberg method (α < 0.05). The code for the analysis can be found here: https://github.com/tnaake/SMARTCARE_preanalytical_processing/tree/main/MLM/metabolomics and https://github.com/tnaake/SMARTCARE_preanalytical_processing/tree/main/MLM/proteomics.

### Pathway analysis of proteomic data

Pathway sets were obtained from the hallmark pathway set collection of the Molecular Signatures Database (MSigDB) of the Broad Institute, along with gene symbol identifiers. Pathway enrichment analysis was performed using the FGSEA R package (version: fgsea_1.18.0), using the proteomic t-values (from “stability” analysis using mixed linear models part) as input statistics. We set the number of permutations to 10,000 and only considered pathway sets with at least 10 protein members. All codes for this part are available at: https://github.com/saezlab/SMARTCARE_pilot_serum_prot_metab.

### Proteomic and metabolomic signatures of plasma and serum samples

The pre-analytical quality signatures were made by selecting the top 20 *p*-values from the LIMMA differential analysis output for serum and plasma samples stored for 8 h compared to 0 h on ice (4°C) or at RT. The contamination signatures were taken from Geyer et al. (2019). In order to estimate the normalized enrichment score, we used the weighted mean method from the decoupleR R package. The weights were either the t-values of the limma differential analysis for the storage quality signatures or the −1*t-value differences for the coagulation signature from Geyer et al. (2019) or the one otherwise (for erythrocyte and platelet contamination since no continuous weights were available). The signatures and method to estimate scores for plasma and serum samples are provided in the form of an open-source R package that can be downloaded here: https://github.com/saezlab/plasmaContamination. The code for the analysis of the sample and computing the scores can be found here: https://github.com/saezlab/SMARTCARE_pilot_serum_prot_metab.

## Data Availability

The original contributions presented in the study are included in the article/Supplementary Materials; further inquiries can be directed to the corresponding author.

## References

[B1] Argelaguet R. Velten B. Arnol D. Dietrich S. Zenz T. Marioni J. C. (2018). Multi‐omics factor analysis—a framework for unsupervised integration of multi‐omics data sets. Mol. Syst. Biol. 14, e8124. 10.15252/msb.20178124 29925568 PMC6010767

[B2] Cao Z. Kamlage B. Wagner-Golbs A. Maisha M. Sun J. Schnackenberg L. K. (2019). An integrated analysis of metabolites, peptides, and inflammation biomarkers for assessment of preanalytical variability of human plasma. J. Proteome Res. 18 (6), 2411–2421. 10.1021/acs.jproteome.8b00903 31074987 PMC8477653

[B3] Daniels J. R. Cao Z. Maisha M. Schnackenberg L. K. Sun J. Pence L. (2019). Stability of the human plasma proteome to pre-analytical variability as assessed by an aptamer-based approach. J. Proteome Res. 18 (10), 3661–3670. 10.1021/acs.jproteome.9b00320 31442052 PMC8480202

[B4] Ferreira D. L. S. Maple H. J. Goodwin M. Brand J. S. Yip V. Min J. L. (2019). The effect of pre-analytical conditions on blood metabolomics in epidemiological studies. Metabolites 9 (4), 64. 10.3390/metabo9040064 30987180 PMC6523923

[B5] Filbin M. R. Mehta A. Schneider A. M. Kays K. R. Guess J. R. Gentili M. (2021). Longitudinal proteomic analysis of severe COVID-19 reveals survival-associated signatures, tissue-specific cell death, and cell-cell interactions. Cell Rep. Med. 2 (5), 100287. 10.1016/j.xcrm.2021.100287 33969320 PMC8091031

[B6] Geyer P. E. Voytik E. Treit P. V. Doll S. Kleinhempel A. Niu L. (2019). Plasma Proteome Profiling to detect and avoid sample‐related biases in biomarker studies. EMBO Mol. Med. 11 (11), e10427. 10.15252/emmm.201910427 31566909 PMC6835559

[B7] Gummesson A. Bjornson E. Fagerberg L. Zhong W. Tebani A. Edfors F. (2021). Longitudinal plasma protein profiling of newly diagnosed type 2 diabetes. EBioMedicine 63, 103147. 10.1016/j.ebiom.2020.103147 33279861 PMC7718461

[B8] Hassis M. E. Niles R. K. Braten M. N. Albertolle M. E. Ewa Witkowska H. Hubel C. A. (2015). Evaluating the effects of preanalytical variables on the stability of the human plasma proteome. Anal. Biochem. 478, 14–22. 10.1016/j.ab.2015.03.003 25769420 PMC4492164

[B9] Heiling S. Knutti N. Scherr F. Geiger J. Weikert J. Rose M. (2021). Metabolite ratios as quality indicators for pre-analytical variation in serum and edta plasma. Metabolites 11 (9), 638. 10.3390/metabo11090638 34564454 PMC8465943

[B10] Kamlage B. Maldonado S. G. Bethan B. Peter E. Schmitz O. Liebenberg V. (2014). Quality markers addressing preanalytical variations of blood and plasma processing identified by broad and targeted metabolite profiling. Clin. Chem. 60 (2), 399–412. 10.1373/clinchem.2013.211979 24305685

[B11] Lippi G. von Meyer A. Cadamuro J. Simundic A. M. (2020). PREDICT: A checklist for preventing preanalytical diagnostic errors in clinical trials. Clin. Chem. Lab. Med. 58 (4), 518–526. 10.1515/cclm-2019-1089 31758854

[B12] Müller T. Kalxdorf M. Longuespee R. Kazdal D. N. Stenzinger A. Krijgsveld J. (2020). Automated sample preparation with SP 3 for low‐input clinical proteomics. Mol. Syst. Biol. 16 (1), e9111. 10.15252/msb.20199111 32129943 PMC6966100

[B13] Naake T. Huber W. (2022). MatrixQCvis: shiny-based interactive data quality exploration for omics data. Bioinformatics 38 (4), 1181–1182. 10.1093/bioinformatics/btab748 PMC8796383 34788796

[B14] Pasella S. Baralla A. Canu E. Pinna S. Vaupel J. Deiana M. (2013). Pre-analytical stability of the plasma proteomes based on the storage temperature. Proteome Sci. 11 (1), 10. 10.1186/1477-5956-11-10 23518135 PMC3658880

[B15] Ryu H. M. Kim Y. J. Oh E. J. Oh S. H. Choi J. Y. Cho J. H. (2016). Hypoxanthine induces cholesterol accumulation and incites atherosclerosis in apolipoprotein E-deficient mice and cells. J. Cell. Mol. Med. 20 (11), 2160–2172. 10.1111/jcmm.12916 27396856 PMC5082407

[B16] Sindelar M. Stancliffe E. Schwaiger-Haber M. Anbukumar D. S. Adkins-Travis K. Goss C. W. (2021). Longitudinal metabolomics of human plasma reveals prognostic markers of COVID-19 disease severity. Cell Rep. Med. 2 (8), 100369. 10.1016/j.xcrm.2021.100369 34308390 PMC8292035

[B17] Stevens V. L. Hoover E. Wang Y. Zanetti K. A. (2019). Pre-analytical factors that affect metabolite stability in human urine, plasma, and serum: A review. Metabolites 9, 156. 10.3390/metabo9080156 31349624 PMC6724180

[B18] Tsonaka R. Signorelli M. Sabir E. Seyer A. Hettne K. Aartsma-Rus A. (2020). Longitudinal metabolomic analysis of plasma enables modeling disease progression in Duchenne muscular dystrophy mouse models. Hum. Mol. Genet. 29 (5), 745–755. 10.1093/hmg/ddz309 32025735 PMC7104681

[B19] Tuck M. Turgeon D. K. Brenner D. E. (2019). “Serum and plasma collection: Preanalytical variables and standard operating procedures in biomarker research,” in Proteomic and metabolomic approaches to biomarker discovery. 2nd edn. (Netherlands: Elsevier). 10.1016/B978-0-12-818607-7.00005-0

[B20] Wagner-Golbs A. Neuber S. Kamlage B. Christiansen N. Bethan B. Rennefahrt U. (2019). Effects of long-term storage at –80 °C on the human plasma metabolome. Metabolites 9 (5), 99. 10.3390/metabo9050099 31108909 PMC6572224

[B21] Yin P. Lehmann R. Xu G. (2015). Effects of pre-analytical processes on blood samples used in metabolomics studies. Anal. Bioanal. Chem. 407, 4879–4892. 10.1007/s00216-015-8565-x 25736245 PMC4471316

