# Supplementary material for: Pre-analytical processing of plasma and serum samples for combined proteome and metabolome analysis
Source: Front Mol Biosci. 2022 Dec 20;9:961448. doi: 10.3389/fmolb.2022.961448 (PMC9808085; doi:10.3389/fmolb.2022.961448)
Supplement: Supplementary file 2 [file DataSheet1.pdf]

Pre-analytical processing of plasma and serum samples for combined proteome and metabolome analysis - Supplementary Information

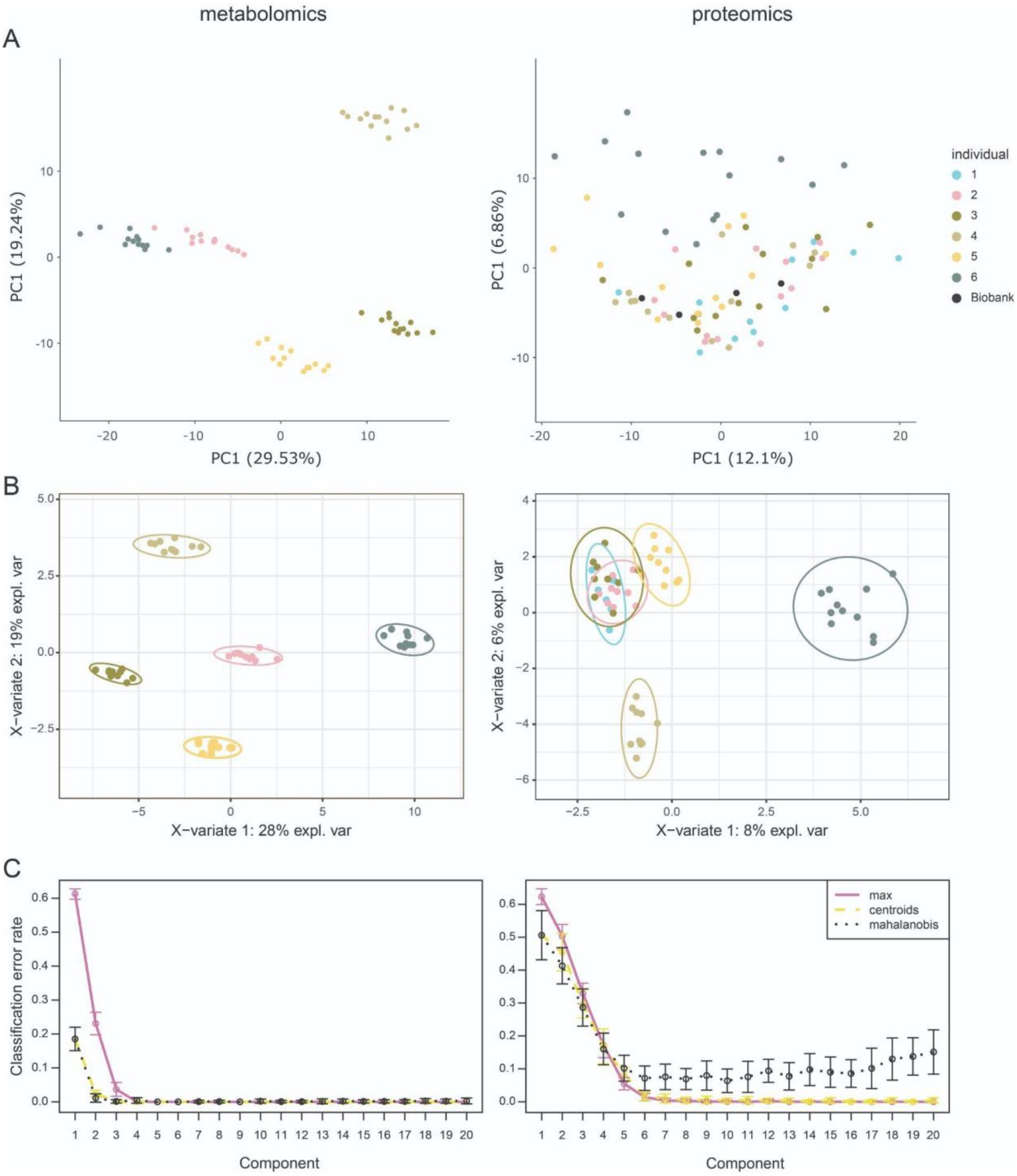

Supplementary Figure 1: Individual-specific effects in the metabolomics and proteomics data set. A: Principal component analysis for metabolomics and proteomics data

set identify individual-specific effects driving the variation within the individual data sets. The effect is more pronounced for the metabolomics data set. Biobank data points refer to long-term reference proteomics plasma samples. B: Sparse partial least squares - discriminant analysis classification using the set of features shown in Supplementary Table 1. The set of features leads to clear separation of individuals in the case of metabolomics, while for the proteomics data set a clear separation is not possible, indicating that it is more difficult to predict the individual based on proteins than on metabolites. C: Classification error rate of Partial least squares - discriminant analysis. Both analyses yield low classification error rates, indicating in general a separability according to the individuals.

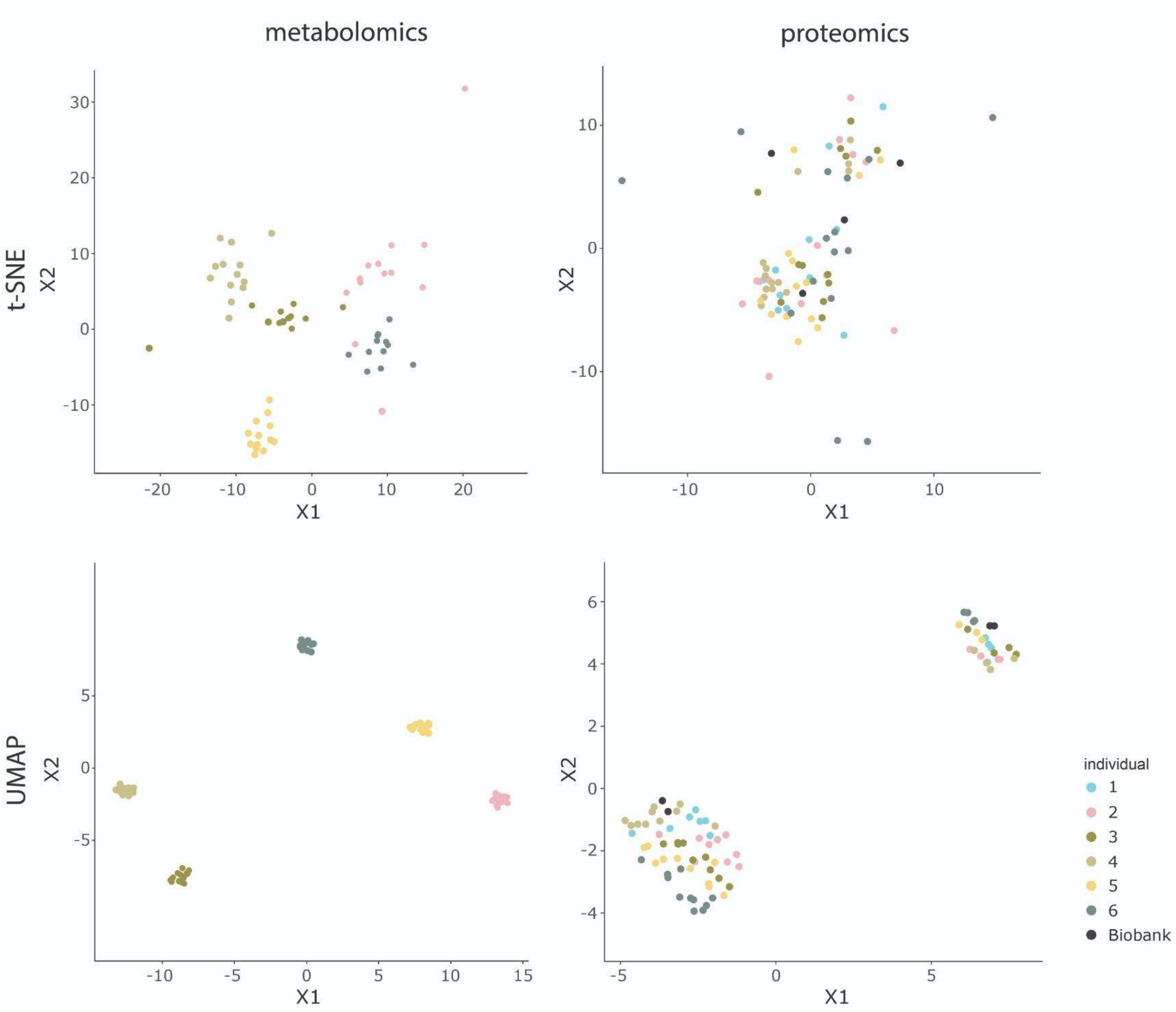

Supplementary Figure 2: Dimension reduction analysis by t-SNE and UMAP of the metabolomics and proteomics data set. t-SNE and UMAP reveals for the metabolomics data set individual-specific effects, while for the proteomics data set t-SNE and UMAP separate the data according to plasma and serum. Biobank data points refer to long-term reference proteomics plasma samples.

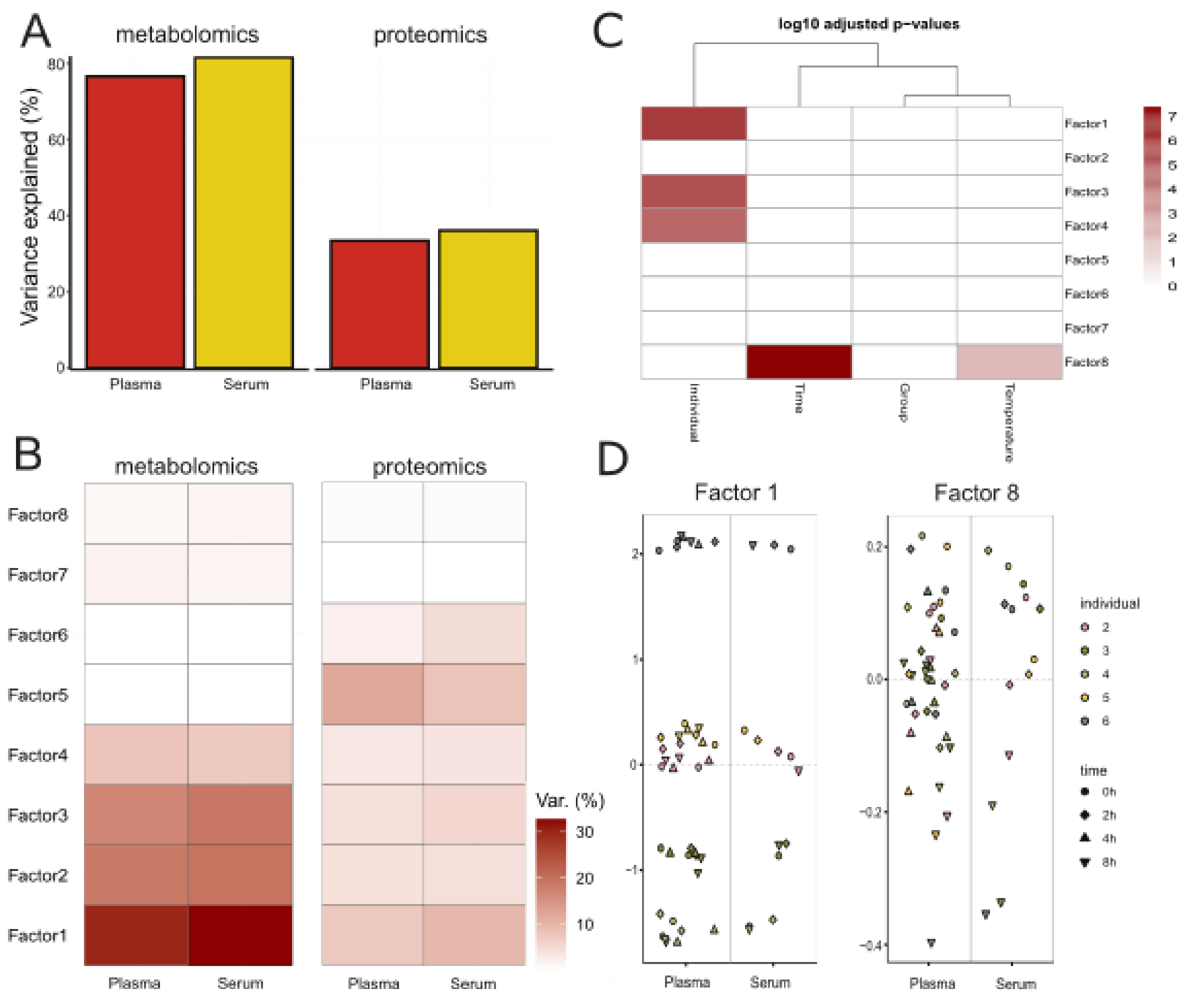

Supplementary Figure 3: Multi-omics factor analysis (MOFA) of the metabolomics and proteomics data set. MOFA infers (hidden) factors that explain biological and technical sources of variability. The factors capture major sources of variation across the proteomics and metabolomics data sets (Argelaguet *et al.*, 2018)). The MOFA model consisted of eight orthogonal axes of heterogeneity (factors 1 to 8) using the 40 and 14 joint samples for metabolomics and proteomics from plasma and serum. A: Variance explained for the metabolomics and proteomics data set for the plasma and serum groups. The fitted model explained 76.6 and 81.5% (R^2_{total}) of the variance in plasma and serum for the metabolomics data set, while for the proteomics data set, it explained 33.36% and 36.1% (R^2_{total}) of the variance in plasma and serum, respectively. B: Factor-wise explanation of variance. The metabolomics data set explains for most of the factors more variance than the proteomics data set. Factor 1 explains 29.9% and 7.5% (R^2_{total}) of the variance in plasma and 32.7% and 9.9% in serum for metabolomics and proteomics, respectively. Factor 8 explains 1.1% and 0.27% (R^2_{total}) of the variance in plasma and 1.32% and 0.47% (R^2_{total}) in serum for metabolomics and proteomics, respectively. C: Association test to check for the association of the eight factors with *individual*, *time*, *group* (plasma, serum), and *temperature*. Factors 1, 3, and 4 were

associated with the *individual*, thereby explaining most of the variance within the data sets, and factor 8 was associated with the *time* variable. D: Visualisation of factors 1 and 8 in latent space. Factor 1 is separating the data set according to the *individual*, while factor 8 is separating the data set according to *time* and *temperature*. The variability along the x-axis includes random jitter.

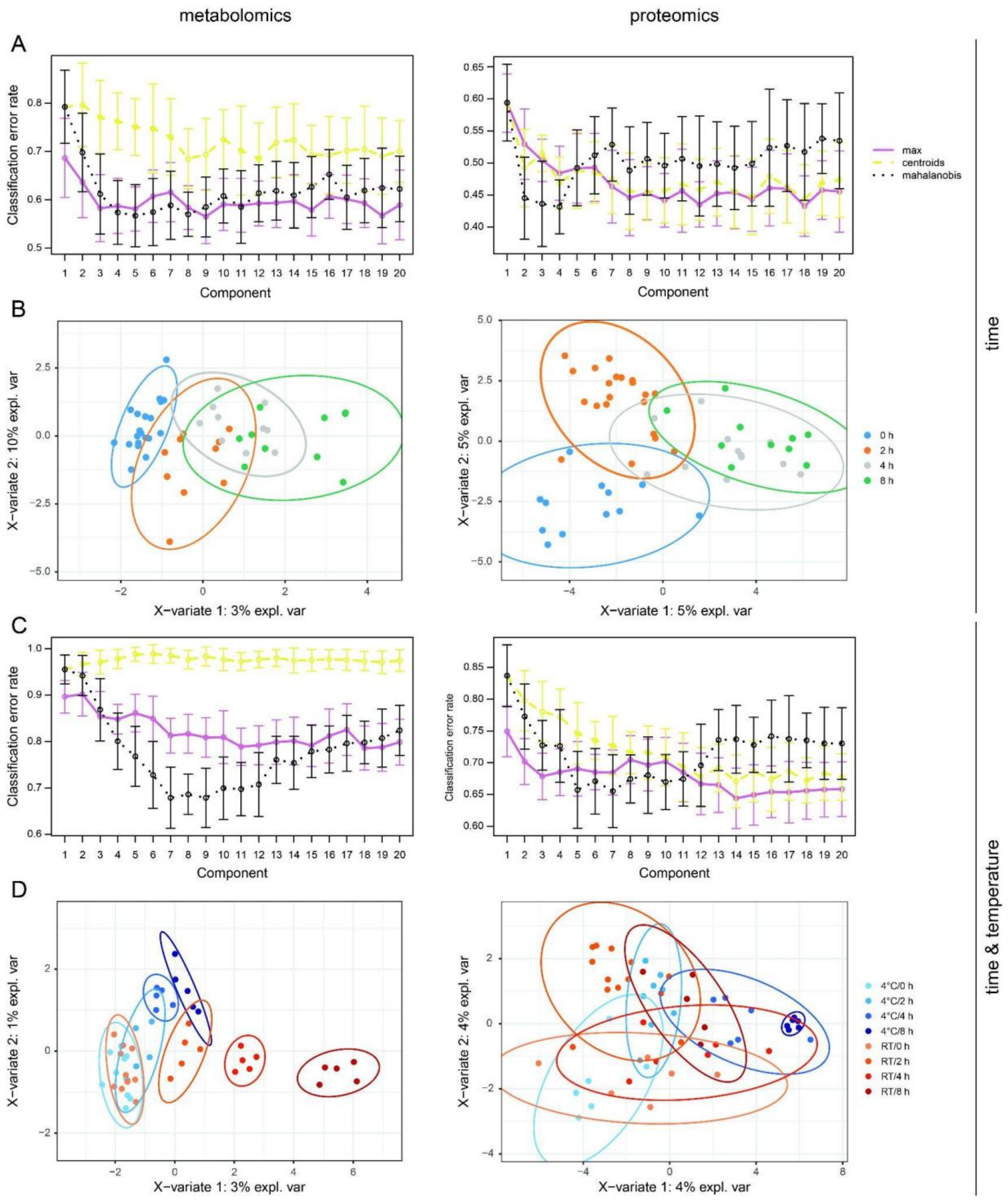

Supplementary Figure 4: Partial least squares - discriminant analysis (PLS-DA) for the metabolomics and proteomics data sets. A: Classification error rates for PLS-DA using the class vector with *time* information. B: Sparse PLS-DA using the class vector with *time* information orders the samples along the time axis. C: Classification error rates for PLS-DA using the class vector with combined information on *time*/*temperature*. D: Sparse PLS-DA using the class vector with combined information on *time*/*temperature*. For the metabolomics data set, there are more distinct clusters for the samples subjected to RT incubation, while the

clusters of the samples at 4°C are less distinct, indicating stronger effects on metabolite levels under RT. The sparse PLS-DA showed a more cluttered picture for the proteomics data set, indicating that the selected protein features are less suitable for classification of the combined *time/temperature* information.

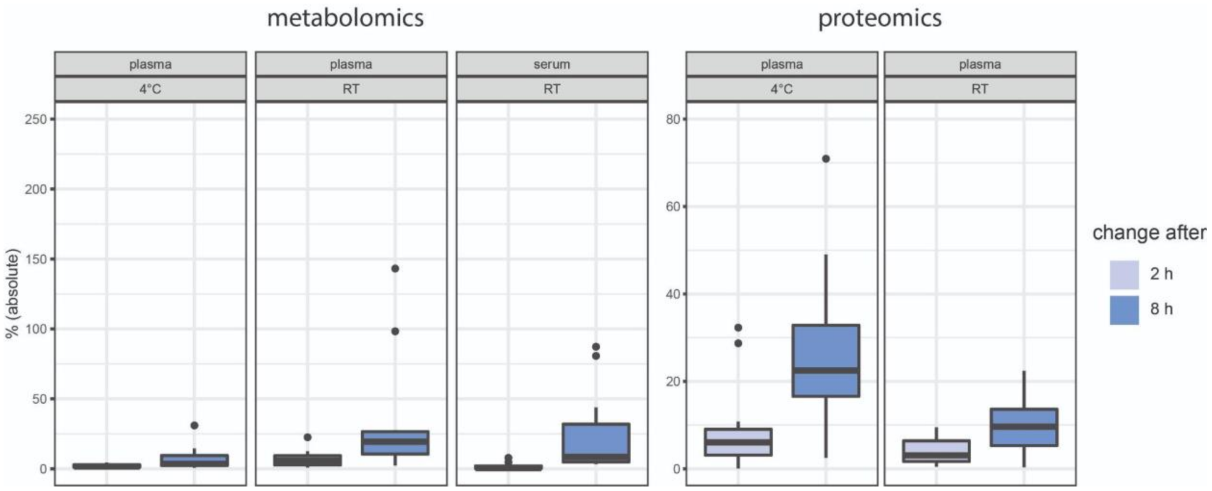

Supplementary Figure 5: Absolute change of significantly changing metabolite and protein levels (in %) for the time points $T_{2\ h}$ and $T_{8\ h}$ compared to $T_{0\ h}$. The intensities at time point 0 h are set to 0 and the changes to the time point 2 h, 4 h, and 8 h are displayed as the mean changes of the individuals (in %). For plasma, the features are included that are significant to the pre-analytical factors *time*, *temperature* or the interaction *time/temperature* (α < 0.05, FDR correction). For serum, the features are included that are significant to the pre-analytical factor *time* (α < 0.05, FDR correction).

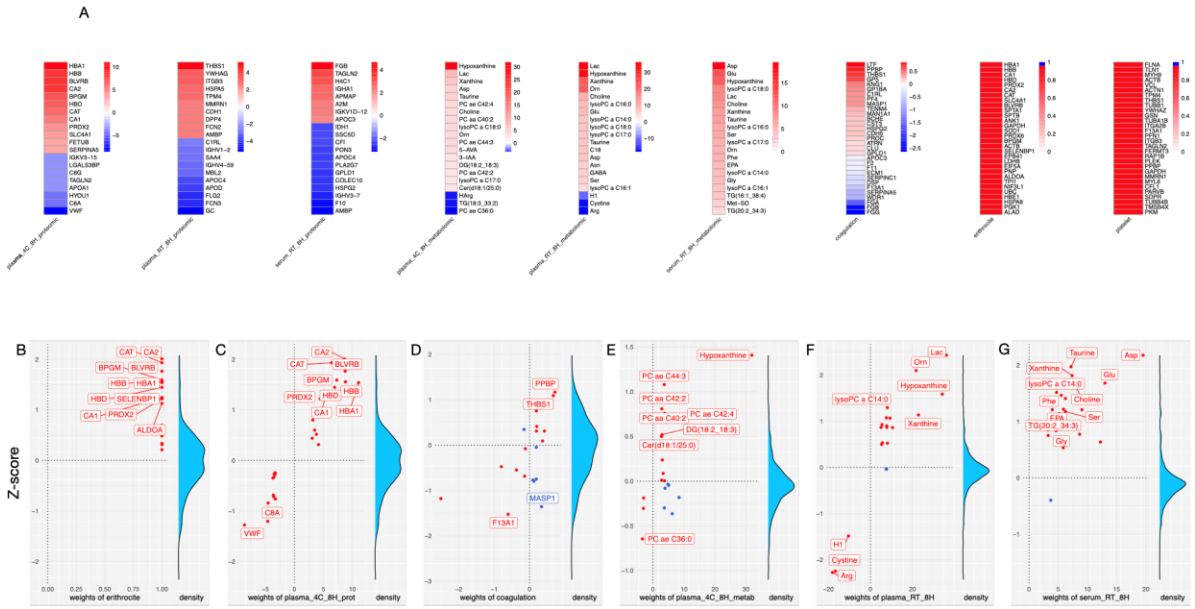

Supplementary Figure 6: Details of signatures included in the plasmaContamination package. A: Proteins and metabolites composing the tested signatures. The colour represents the weight of each feature of the signature, when available. The signature score is computed as a weighted mean of the feature weight * feature measurement, normalised using an empirical distribution of score generated through feature shuffling. B, C, D, E, F, G: Scatter plot of feature weight against feature measurements for the corresponding samples/contrasts (a contrast being the result of a differential analysis between two conditions). B, C, E: relative to plasma samples kept at 4°C for 8 h. D, G: relative to serum samples kept at RT for 8 h. F: relative to plasma samples kept at RT for 8 h. Red and blue colours represent positive and negative contributions to the signature score, respectively. Red corresponds to up/down-regulated features with positive/negative weights, respectively, and blue corresponds to up/down-regulated features with negative/positive weights, respectively. The features that are the furthest away from the (0, 0) coordinate have the highest contribution to the score (contribution of a single feature being defined as weight * measurement).

Supplementary Table 1: Selected features of sparse partial least square - discriminant analysis using the *individual* as the class vector. The intensities of the features are ordered in descending order according to the absolute importance values.

metabolite	Value of selected metabolite	protein	Value of selected protein
PC.ae.C36:5	-0.268500768	SAA2 (P0DJI9)	0.58498936
GDCA	-0.266503328	C9 (P02748)	0.51144060
PC.ae.C34:3	-0.256407863	IGLV3-21 (P80748)	-0.37696364
CE(14:0)	-0.229114859	CRP (P02741)	0.28526630
PC.ae.C36:4	-0.223906293	PON1 (P27169)	-0.23718690
CE(20:4)	-0.223493432	APOC4 (P55056)	-0.18297455
PC.ae.C38:5	-0.218488828	IGLV2-8 (P01709)	0.17191216
TG(20:2_34:1)	0.218144999	CFB (P00751)	0.15198452
PC.aa.C38:4	-0.214187090	F13A1 (P00488)	-0.14917795
TG(20:1_34:2)	0.207074762	GSTO1 (P78417)	0.04788159
PC.ae.C38:4	-0.204103703	FCGBP (Q9Y6R7)	0.04452931
TG(16:0_38:2)	0.191128128	PRAP1 (Q96NZ9;Q96NZ9-2; Q96NZ9-3;Q96NZ9-4)	0.04218922
TG(20:1_34:1)	0.173650357	TTR (P02766)	-0.03921732
CE(17:1)	-0.172349100	C1S (P09871)	-0.01751029
PC.ae.C32:1	-0.170670334	ALDOB (P05062)	-0.01389305
PC.ae.C38:6	-0.167923231		
PC.ae.C40:5	-0.161312384		
HexCer(d18:2/24:0)	-0.159319689		
PC.aa.C36:4	-0.158318121		
TDCA	-0.153837447		
HexCer(d18:1/24:0)	-0.143365272		
TG(16:0_38:3)	0.132706459		
DCA	-0.119516370		
PC.aa.C40:5	-0.110879600		
TG(18:1_36:1)	0.109261217		
GUDCA	0.101513121		
lysoPC.a.C20:4	-0.094747816		
PC.aa.C38:0	-0.086969074		
PC.ae.C34:2	-0.086936894		
TG(18:0_36:3)	0.081121569		
PC.ae.C40:6	-0.080362096		
CE(22:5)	-0.079775441		
TG(18:0_36:2)	0.077673974		
FA(18:2)	0.074525414		
CE(18:0)	-0.069044645		
TG(18:1_34:2)	0.046089288		
HexCer(d18:1/23:0)	-0.043501804		
SM.C18:1	-0.040112031		
PC.aa.C38:5	-0.034541815		

HipAcid	-0.033557136		
CE(15:0)	-0.027731165		
Kynurenine	0.027723492		
CE(14:1)	-0.026967113		
TrpBetaine	0.026213738		
PC.aa.C40:4	-0.024257807		
lysoPC.a.C28:1	-0.022013954		
PC.ae.C32:2	-0.012042735		
CE(18:1)	-0.003493789		
CE(16:1)	-0.001702684		
PC.ae.C34:1	-0.000103610		

Supplementary Table 2: Selected features of sparse partial least square - discriminant analysis using the *time* as the class vector. The features are ordered in descending order according to the absolute importance values.

Metabolite	Value of selected metabolite	Protein	Value of selected protein
Hypoxanthine	0.86500382	HBA1 (P69905)	0.3051776936
Xanthine	0.35585116	ECM1 (Q16610;Q16610-4)	-0.2790432656
Lactate	0.33061249	BPGM (P07738)	0.2728424861
Ornithine	0.12100160	APOA1 (P02647)	-0.2689895678
Asparagine	0.03453019	CAT (P04040)	0.2574125644
		CA2 (P00918)	0.2506049009
		HYOU1 (Q9Y4L1)	-0.2468780420
		SLC4A1 (P02730)	0.2259034325
		FERMT3 (Q86UX7;Q86UX7-2)	0.2201556911
		HBB (P68871)	0.2103457860
		BASP1 (P80723;P80723-2)	-0.2062149021
		HBD (P02042)	0.1863074451
		BLVRB (P30043)	0.1823127035
		CA1 (P00915)	0.1754204972
		YWHAZ (P63104)	0.1705790612
		MPO (P05164;P05164-2; P05164-3)	0.1635279760
		GANAB (Q14697;Q14697-2)	0.1397937894
		GC (P02774)	-0.1394798788
		F2 (P00734)	-0.0955141175
		C8G (P07360)	-0.0934011313
		CFL1 (P23528)	0.0886430466
		PRSS3 (P35030;P35030-2; P35030-3;P35030-4)	0.0864709428
		PROZ (P22891;P22891-2)	0.0792679857
		FN1 (P02751;P02751-3)	-0.0788933328
		HPX (P02790)	-0.0709280449
		VASN (Q6EMK4)	-0.0665273047
		ATRN (O75882-2;O75882-3)	0.0650318704
		PSMA4 (P25789)	-0.0628043092
		KRT8 (P05787)	-0.0606475289
		MDH1 (P40925;P40925-2)	0.0599833383
		IGKV3-20 (P04206)	-0.0587456769
		EEF1A1 (P68104;Q5VTE0)	-0.0584839501
		PRDX2 (P32119)	0.0578632531

		ITIH2 (P19823)	0.0550239670
		PTPRG (P23470;P23470-2)	0.0438356128
		EFEMP1 (Q12805;Q12805-2;Q12805-3;Q12805-4)	0.0429530829
		ITIH3 (Q06033;Q06033-2)	0.0423345831
		ITGB3 (P05106)	0.0414866691
		HNRNPK (P61978;P61978-2; P61978-3)	-0.0407078585
		ALDOA (P04075)	0.0406151070
		GP1BB (P13224;P13224-2)	0.0405218107
		TPM3 (P06753-2)	0.0397427929
		IGLC2 (P0CG05)	-0.0388841132
		IGHG1 (P01857)	0.0387823216
		TFRC (P02786)	0.0383223128
		VWF (P04275)	-0.0332107407
		IGHV3-7 (P80419)	-0.0331100294
		MRC1 (P22897)	0.0325502649
		HSPA8 (P11142;P11142-2)	0.0324084800
		PRDX1 (Q06830)	-0.0292653697
		PGK1 (P00558)	0.0266566339
		F12 (P00748)	0.0246458975
		FGL1 (Q08830)	0.0237648829
		CFI (P05156)	0.0230229098
		CLU (P10909;P10909-2; P10909-4;P10909-5)	0.0223664138
		PRDX6 (P30041)	0.0182668762
		IGLV6-57 (P06318)	0.0160594551
		FLT4 (P35916;P35916-1; P35916-3)	-0.0159933947
		TLN1 (Q9Y490)	0.0143133844
		APOA4 (P06727)	-0.0138128179
		MASP1 (P48740-2;P48740-4)	0.0130026995
		NME2 (P22392;P22392-2)	0.0122881335
		ITGA2B (P08514;P08514-2)	0.0109002438
		AHSG (P02765)	-0.0106803269
		ANGPTL3 (Q9Y5C1)	-0.0102464019
		AFM (P43652)	-0.0091812995
		DPP4 (P27487)	0.0081835342
		IGKV1D-33 (P01593)	-0.0055046478
		KNG1 (P01042-2)	0.0042764859
		DSG2 (Q14126)	0.0037493018

		C8A (P07357)	-0.0035858940
		NFX1 (Q12986;Q12986-2)	-0.0031747150
		DSC1 (Q08554;Q08554-2)	-0.0030473481
		HLA-A (P01891;P01892;P04439;P05534;P10314;P10316;P13746;P13746-2;P16188;P16189;P16190;P18462;P30443;P30447;P30450;P30453;P30455;P30456;P30457;P30459;P30508;P30512;Q29960;Q29960-2;Q95604)	0.0013763727
		SAA4 (P35542)	-0.0002944848

Supplementary Table 3: Selected features for sparse partial least square – discriminant analysis using *temperature* as the class vector. The features are ordered in descending order according to the absolute importance values.

Metabolite	Value of selected metabolite	Protein	Value of selected protein
Lactate	-0.60085947	THBS1 (P07996)	0.6521617731
Ornithine	-0.54910269	PF4 (P02776;P10720)	0.6318854387
Xanthine	-0.36905947	VWF (P04275)	0.3135767399
Arginine	0.28784931	AMBP (P02760)	0.1908734707
Cystine	0.27148684	PPBP (P02775)	0.1750974544
Aconitic acid	-0.20830526	FN1 (P02751;P02751-3)	0.0964168595
Hexosyl ceramide (d18:2/16:0)	0.03599758	TKT (P29401)	-0.0231791401
		KRT6B (P04259)	-0.0122508252
		GP1BB (P13224;P13224-2)	-0.0002753722

Supplementary Table 4: Selected features for sparse partial least square – discriminant analysis using the combined factor *time/temperature* as the class vector. The features are ordered in descending order according to the absolute importance values.

**Metabolite**	**Value of selected metabolite**	**protein**	**Value of selected protein**
Lactate	0.497906200	HBA1 (P69905)	0.390132770
Xanthine	0.482245661	BPGM (P07738)	0.347052972
Hypoxanthine	0.385472090	CAT (P04040)	0.336819132
Ornithine	0.382345986	CA2 (P00918)	0.295104965
Cystine	-0.315990537	HBB (P68871)	0.259492546
Arginine	-0.299029707	HBD (P02042)	0.250791582
lysoPC.a.C14:0	0.117475537	BLVRB (P30043)	0.235249620
C16	0.109778009	SLC4A1 (P02730)	0.209868590
Choline	0.066216195	CA1 (P00915)	0.202047270
H1	-0.061548518	BASP1 (P80723;P80723-2)	-0.174176982
C18	0.022301374	VWF (P04275)	-0.173046419
Aconitic acid	0.020311751	APOA1 (P02647)	-0.163684244
lysoPC.a.C16:0	0.017172037	HYOU1 (Q9Y4L1)	-0.160338017
Aspartic acid	0.013924487	FN1 (P02751;P02751-3)	-0.159296043
TG(20:1_32:2)	-0.007704635	CFL1 (P23528)	0.147770162
		PRDX2 (P32119)	0.140059427
		FERMT3 (Q86UX7;Q86UX7-2)	0.127839385
		VASN (Q6EMK4)	-0.125158205
		YWHAZ (P63104)	0.090406370
		GANAB (Q14697;Q14697-2)	0.084510361
		EFEMP1 (Q12805;Q12805-2; Q12805-3;Q12805-4)	0.083982491
		ECM1 (Q16610;Q16610-4)	-0.075987107
		PRSS3 (P35030;P35030-2; P35030-3;P35030-4)	0.071483516

		MDH1 (P40925;P40925-2)	0.070693769
		C8G (P07360)	-0.058401316
		F2 (P00734)	-0.033569852
		KRT8 (P05787)	-0.024205527
		ATRN (O75882-2;O75882-3)	0.021001097
		GP1BB (P13224;P13224-2)	0.015455550
		MRC1 (P22897)	0.013485628
		ALDOA (P04075)	0.012840246
		C8A (P07357)	-0.012307833
		GSN (P06396-2)	-0.011774569
		PROZ (P22891;P22891-2)	0.007770302
		EEF1A1 (P68104;Q5VTE0)	-0.001995734

Author contributions

F.C. and H.M.G. designed the experiment. F.C., H.M.G. and N.K.-R. performed the experiment. H.M.G., G.K. and T.M. measured the metabolites and proteins. T.N. and A.D. analysed and interpreted the data set. A.D., H.M.G., T.M., and T.N. wrote the manuscript. E.J. and C.H suggested methods and experiments and provided feedback on the manuscript. B.H., U.K., C.M.-T., S.D., J.S.-R., W.H., R.H., G.P., and J.K. provided feedback to the manuscript.

Conflict of Interest

J.S.-R. reports funding from GSK and Sanofi and fees from Travere Therapeutics and Astex Therapeutics.

Acknowledgements

This work was supported by the German Ministry of Education and Research (BMBF), as part of the National Research Node “Mass spectrometry in Systems Medicine” (MSCoreSys), under the funding code 161L0212. Several figures were created with BioRender.com.

References

Argelaguet, R. *et al.* (2018) ‘Multi-Omics Factor Analysis—a framework for unsupervised integration of multi-omics data sets’, *Molecular Systems Biology*. doi: 10.15252/msb.20178124.

Cao, Z. *et al.* (2019) ‘An Integrated Analysis of Metabolites, Peptides, and Inflammation Biomarkers for Assessment of Preanalytical Variability of Human Plasma’, *Journal of Proteome Research*, 18(6), pp. 2411–2421. doi: 10.1021/acs.jproteome.8b00903.

Daniels, J. R. *et al.* (2019) ‘Stability of the Human Plasma Proteome to Pre-analytical Variability as Assessed by an Aptamer-Based Approach’, *Journal of Proteome Research*, 18(10), pp. 3661–3670. doi: 10.1021/acs.jproteome.9b00320.

Ferreira, D. L. S. *et al.* (2019) ‘The effect of pre-analytical conditions on blood metabolomics in epidemiological studies’, *Metabolites*, 9(4). doi: 10.3390/metabo9040064.

Filbin, M. R. *et al.* (2021) ‘Longitudinal proteomic analysis of severe COVID-19 reveals survival-associated signatures, tissue-specific cell death, and cell-cell interactions’, *Cell Reports Medicine*, 2(5). doi: 10.1016/j.xcrm.2021.100287.

Geyer, P. E. *et al.* (2019) ‘Plasma Proteome Profiling to detect and avoid sample-related biases in biomarker studies’, *EMBO Molecular Medicine*, 11(11). doi: 10.15252/emmm.201910427.

Gummesson, A. *et al.* (2021) ‘Longitudinal plasma protein profiling of newly diagnosed type 2 diabetes’, *EBioMedicine*, 63. doi: 10.1016/j.ebiom.2020.103147.

Hassis, M. E. *et al.* (2015) ‘Evaluating the effects of preanalytical variables on the stability of the human plasma proteome’, *Analytical Biochemistry*. Elsevier Inc., 478, pp. 14–22. doi: 10.1016/j.ab.2015.03.003.

Heiling, S. *et al.* (2021) ‘Metabolite ratios as quality indicators for pre-analytical variation in serum and edta plasma’, *Metabolites*, 11(9). doi: 10.3390/metabo11090638.

Kamlage, B. *et al.* (2014) ‘Quality markers addressing preanalytical variations of blood and plasma processing identified by broad and targeted metabolite profiling’, *Clinical Chemistry*, 60(2), pp. 399–412. doi: 10.1373/clinchem.2013.211979.

Lippi, G. *et al.* (2020) ‘PREDICT: A checklist for preventing preanalytical diagnostic errors in clinical trials’, *Clinical Chemistry and Laboratory Medicine*, 58(4), pp. 518–526. doi:

10.1515/cclm-2019-1089.

Müller, T. *et al.* (2020) ‘Automated sample preparation with SP 3 for low-input clinical proteomics’, *Molecular Systems Biology*, 16(1). doi: 10.15252/msb.20199111.

Naake, T. and Huber, W. (2022) ‘MatrixQCvis: shiny-based interactive data quality exploration for omics data’, *Bioinformatics*, 38(4), pp. 1181–1182. doi: 10.1093/bioinformatics/btab748.

Pasella, S. *et al.* (2013) ‘Pre-analytical stability of the plasma proteomes based on the storage temperature’, *Proteome Science*, 11(1), pp. 1–10. doi: 10.1186/1477-5956-11-10.

Ryu, H. M. *et al.* (2016) ‘Hypoxanthine induces cholesterol accumulation and incites atherosclerosis in apolipoprotein E-deficient mice and cells’, *Journal of Cellular and Molecular Medicine*, 20(11), pp. 2160–2172. doi: 10.1111/jcmm.12916.

Sindelar, M. *et al.* (2021) ‘Longitudinal metabolomics of human plasma reveals prognostic markers of COVID-19 disease severity’, *Cell Reports Medicine*, 2(8). doi: 10.1016/j.xcrm.2021.100369.

Stevens, V. L. *et al.* (2019) ‘Pre-analytical factors that affect metabolite stability in human urine, plasma, and serum: A review’, *Metabolites*. doi: 10.3390/metabo9080156.

Tsonaka, R. *et al.* (2020) ‘Longitudinal metabolomic analysis of plasma enables modeling disease progression in Duchenne muscular dystrophy mouse models’, *Human Molecular Genetics*, 29(5), pp. 745–755. doi: 10.1093/hmg/ddz309.

Tuck, M., Turgeon, D. K. and Brenner, D. E. (2019) *Serum and plasma collection: Preanalytical variables and standard operating procedures in biomarker research*. 2nd edn, *Proteomic and Metabolomic Approaches to Biomarker Discovery*. 2nd edn. Elsevier Inc. doi: 10.1016/B978-0-12-818607-7.00005-0.

Wagner-Golbs, A. *et al.* (2019) ‘Effects of long-term storage at –80 °C on the human plasma metabolome’, *Metabolites*, 9(5). doi: 10.3390/metabo9050099.

Yin, P., Lehmann, R. and Xu, G. (2015) ‘Effects of pre-analytical processes on blood samples used in metabolomics studies’, *Analytical and Bioanalytical Chemistry*, pp. 4879–4892. doi: 10.1007/s00216-015-8565-x.
